# Piperacillin Sodium and Tazobactam Sodium‐Induced Neutropenia and Thrombocytopenia: A Case Report

**DOI:** 10.1002/ccr3.71062

**Published:** 2025-10-16

**Authors:** Hongyu Zhang, Kaile Wu, Ka Liang, Zheng Peng, Ping Yi

**Affiliations:** ^1^ Department of Clinical Laboratory The Third Affiliated Hospital of Guangxi University of Chinese Medicine, Liuzhou Traditional Chinese Medical Hospital, The Third Clinical Faculty of Guangxi University of Chinese Medicine Liuzhou Guangxi China; ^2^ Department of Gastroenterology The Third Affiliated Hospital of Guangxi University of Chinese Medicine, Liuzhou Traditional Chinese Medical Hospital, The Third Clinical Faculty of Guangxi University of Chinese Medicine Liuzhou Guangxi China

**Keywords:** case report, neutropenia, piperacillin, tazobactam drug combination, thrombocytopenia

## Abstract

Piperacillin sodium/tazobactam sodium (PIP‐TAZ) is a commonly used antibiotic for various infections. While generally well‐tolerated, it has been associated with rare cases of neutropenia and thrombocytopenia. This case report highlights the potential for PIP‐TAZ to cause these adverse effects, emphasizing the importance of vigilant monitoring and timely intervention. A 79‐year‐old female with chronic renal failure and end‐stage renal disease developed neutropenia and thrombocytopenia during treatment with PIP‐TAZ for a suspected lung infection. Her neutrophil count plummeted from 11.17 × 10^9^/L to 0.37 × 10^9^/L, while her platelet count dropped from 282 × 10^9^/L to 4 × 10^9^/L. Despite interventions including platelet transfusions, the counts only normalized after discontinuing PIP‐TAZ.P2P4. The patient's clinical course and response to discontinuation of PIP‐TAZ suggest a likely causal relationship between the drug and her hematological abnormalities. Differential diagnoses, including severe infection, disseminated intravascular coagulation, and hematological malignancies, were ruled out through clinical examination and laboratory tests. The exact mechanism by which PIP‐TAZ causes neutropenia and thrombocytopenia remains unclear but may involve immune‐mediated reactions, direct drug toxicity, individual differences, and potential drug interactions in patients with renal impairment. Preventative measures include thorough pre‐treatment evaluation, close monitoring of complete blood counts, individualized medication regimens, and prompt discontinuation upon suspicion of adverse effects. This case report underscores the need for clinicians to be aware of the potential hematological adverse effects of PIP‐TAZ, particularly in patients with chronic diseases or renal insufficiency. Regular monitoring, prompt recognition, and timely intervention are crucial for ensuring patient safety and minimizing the risk of serious complications.

**Summary**

- This case highlights the potential for piperacillin sodium and tazobactam sodium for injection (PIP‐TAZ) to cause neutropenia and thrombocytopenia, emphasizing the importance of close monitoring and early intervention.

## 1 Introduction

Piperacillin sodium and tazobactam sodium for injection (PIP‐TAZ) is a combined antibiotic therapy consisting of the penicillin‐based drug piperacillin paired with the β‐lactamase inhibitor tazobactam. This combination works primarily by disrupting the synthesis of bacterial cell walls and inhibiting the formation of cell membranes, which results in a bactericidal outcome [1]. The drug demonstrates good antibacterial activity against a variety of Gram‐positive and Gram‐negative aerobic and anaerobic bacteria and is indicated for a range of infectious diseases, including community‐ and hospital‐acquired pneumonia, urinary tract infections, intra‐abdominal infections, skin and soft tissue infections, bacterial sepsis, and inflammatory diseases of the female reproductive system (such as endometritis and pelvic inflammatory disease). In clinical use, PIP‐TAZ may also cause some adverse reactions, commonly including allergic reactions, nausea, vomiting, diarrhea, jaundice, renal insufficiency, and blood system‐related adverse reactions [2]. Multiple studies have indicated that PIP‐TAZ‐associated hematologic toxicity may be related to direct drug toxicity, immune‐mediated mechanisms, or drug interactions, with a higher incidence particularly observed in critically ill or immunocompromised populations [3, 4, 5]. In such patients experiencing PIP‐TAZ‐related hematologic adverse reactions, the clinical manifestations may be more severe, necessitating careful consideration when balancing anti‐infective efficacy against potential drug toxicity. The exact mechanism by which PIP‐TAZ induces neutropenia and thrombocytopenia remains elusive, potentially stemming from either the direct toxic impact of the medication or immune‐mediated responses. This case report aims to describe a rare occurrence of neutropenia and thrombocytopenia in a patient receiving treatment with PIP‐TAZ, and to raise awareness among clinicians of the potential adverse reactions of this drug.

## 2 Case History

A 79‐year‐old woman with chronic renal failure and end‐stage renal disease is currently receiving maintenance hemodialysis. She also has a history of hypertension. A week ago, the patient presented with respiratory symptoms, including cough, sputum production, and difficulty breathing, indicating a potential lung infection. The significant increase in the neutrophil count further supported this diagnosis. During the treatment process, she experienced severe hyperkalemia, and her electrocardiogram indicated a potential cardiac risk, necessitating emergency medication intervention and hemodialysis. For anti‐infection treatment, PIP‐TAZ was initially selected as empirical therapy, and a sputum culture was performed to identify the pathogen. When using PIP‐TAZ via intravenous infusion, the patient is administered a dose of 2 g every 12 h. The patient's neutrophil and platelet counts significantly decreased during treatment, raising concerns about drug‐induced bone marrow suppression. Subsequently, the patient was treated to increase platelet levels and received platelet transfusions, while PIP‐TAZ was discontinued. After the drug was discontinued, the patient's platelet count recovered. At the time of discharge, the patient's condition had improved, with reduced swelling of the extremities, occasional mild cough, and sputum at night, but no other severe symptoms. Her quality of life and daily activities were not significantly affected.

## 3 Methods

### 3.1 Investigations

We used the Mindray automatic hematology analyzer (Mindray Co., China) to measure neutrophil and platelet counts. If the platelet count was less than 50 × 10^9^/L, manual verification was performed to rule out pseudothrombocytopenia. The patient's blood test upon admission showed a significant increase in the count of neutrophils to 11.17 × 10^9^/L, which is well above the normal range (1.80–6.30 × 10^9^/L), possibly indicating the presence of infection or inflammation. The platelet count was 282 × 10^9^/L, within the normal range (140–440 × 10^9^/L), while the C‐reactive protein (CRP) level was exceptionally high, exceeding 200.00 mg/L, far above the normal upper limit of 8 mg/L, further supporting the diagnosis of infection or inflammation. In addition, the patient was admitted to the hospital for lung imaging, which was suggestive of cloudy flocculent shadows in the lungs. The results of the computed tomography (CT) scan also indicated that the patient had a lung infection.

After 5 days of treatment, the neutrophil count decreased to 8.71 × 10^9^/L, the platelet count slightly dropped to 260 × 10^9^/L, and the CRP level decreased to 34.39 mg/L, indicating that the infection or inflammation had been controlled to some extent.

However, on the 10th day of review, the neutrophil count sharply decreased to 0.37 × 10^9^/L, and the platelet count also dropped to 4 × 10^9^/L, with the CRP level falling to 29.27 mg/L. The significant decrease in platelet count prompted the physician to order two therapeutic doses of single‐donor platelet transfusion.

The review on the 11th day showed a slight rebound in the neutrophil count to 1.39 × 10^9^/L, and a small increase in the platelet count to 5 × 10^9^/L. At the same time, the prothrombin time (PT) was prolonged to 14.4 s, exceeding the normal range (11–13 s), and the fibrinogen level increased to 4.31 g/L, above the normal upper limit (2–4 g/L). Hematological examination found that the red blood cells were of uneven size, with some central pale areas enlarged; no primitive immature cells were seen, and the platelet count was low.

After the platelet transfusion, the platelet count did not significantly increase, suspected to be due to drug interference; the physician immediately discontinued the suspicious medication. The review on the 14th day showed that the neutrophil count had recovered to 4.09 × 10^9^/L, the platelet count had significantly increased to 138 × 10^9^/L, and the CRP level further decreased to 12.17 mg/L, but it was still slightly above the normal upper limit.

On the 17th day, when the patient was discharged, the neutrophil count was 6.12 × 10^9^/L, and the platelet count was 259 × 10^9^/L, both back within the normal range. These data indicate that the patient's infection or inflammation was effectively controlled, the platelet count was also restored, and the treatment was successful.

### 3.2 Differential Diagnosis

In diagnosing a patient with a sudden drop in neutrophil and platelet counts, a thorough differential diagnosis is key, including assessing medication‐induced bone marrow suppression, severe infection, and disseminated intravascular coagulation (DIC), while also ruling out heparin‐induced thrombocytopenia, nutritional deficiencies, autoimmune conditions, and hematological cancers [6]. The patient's decreasing CRP suggests infection control, and normal prothrombin time and blood morphology exclude DIC and malignancies. Normal immunoglobulin and complement levels, the absence of autoimmune symptoms, and no heparin use point away from immune‐mediated cytopenia and heparin‐induced thrombocytopenia. Thus, PIP‐TAZ is suspected as the cause of the cytopenias based on test results, clinical presentation, and treatment history.

### 3.3 Treatment

**FIGURE 1:**
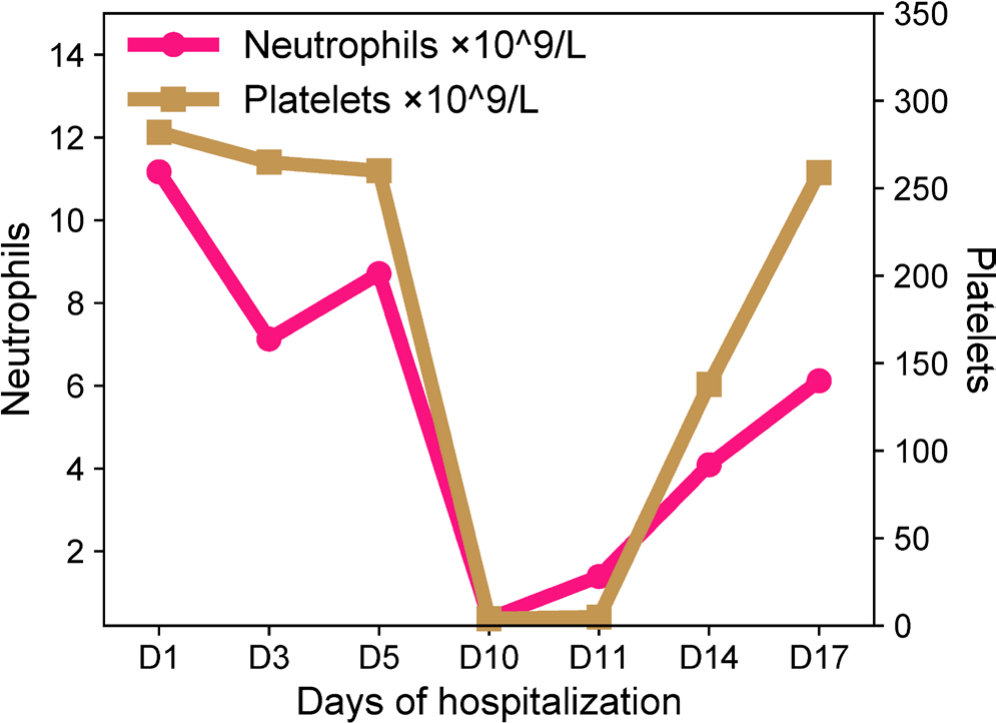
Temporal trends in neutrophil and platelet counts during Piperacillin sodium/tazobactam sodium (PIP‐TAZ) therapy and post‐discontinuation. Neutrophil count and platelet count are plotted against treatment days (Days 1–17). PIP‐TAZ was administered from day 1 to day 10, with a platelet transfusion on day 10, followed by discontinuation of the treatment on day 11. Reference ranges: Neutrophils 1.8–6.3 × 10^9^/L; platelets 140–440 × 10^9^/L.

During the patient's hospital stay, we observed a significant decrease in neutrophil and platelet counts. After analysis, we strongly suspected that this phenomenon was related to the drug the patient was using, PIP‐TAZ. On the 10th day, the patient's platelet and neutrophil counts were severely reduced, and we took measures to transfuse 2 units of platelets. However, during the re‐examination on the 11th day, the count results still showed no significant improvement, which led us to be more convinced that the drug might have an inhibitory effect on the bone marrow. Therefore, we immediately discontinued the suspected drug, PIP‐TAZ. Subsequently, on the 14th day, the patient's neutrophil and platelet counts recovered to 4.09 × 10^9^/L and 138 × 10^9^/L, respectively. This trend (as shown in Figure 1) further confirmed our speculation: the side effects of PIP‐TAZ caused a reduction in the patient's neutrophils and platelets. Nursing care after discontinuation of medication consisted mainly of close monitoring of the patient's vital signs. Other nursing services were no different from those previously provided.

## 4 Results

This case report emphasizes that significant hematological adverse reactions, such as neutropenia and thrombocytopenia, can occur even with short courses and therapeutic doses of PIP‐TAZ. Therefore, regular monitoring of complete blood cell counts is necessary for patients with chronic diseases and/or renal insufficiency, especially when using such antibiotics. Rapid identification and cessation of the suspected drug are crucial once a sharp change in hematological parameters is observed. Additionally, individualized treatment plans are essential to reduce the risk of adverse reactions. Increasing vigilance, education, communication, and multidisciplinary collaboration are effective strategies to ensure patient safety and reduce the incidence of serious adverse events.

## 5 Discussion

There are also reports in the past literature indicating that piperacillin‐tazobactam can induce neutropenia and thrombocytopenia, and these reports have explored the possible factors involved [7, 8, 9]. A case report was made of a 79‐year‐old female patient who developed neutropenia and thrombocytopenia after receiving treatment with PIP‐TAZ. The patient's blood cell counts rapidly returned to normal after discontinuing the medication, suggesting that PIP‐TAZ may be the primary cause of these adverse reactions.

PIP‐TAZ is a commonly used antibiotic, but in rare cases, it can lead to neutropenia and thrombocytopenia [8, 10]. The exact mechanism by which PIP‐TAZ causes neutropenia and thrombocytopenia is still unclear, but it may involve the following factors:

1. *Immune‐mediated reactions*: Piperacillin or tazobactam may bind to antibodies in the patient's body, forming drug‐antibody complexes that can directly damage neutrophils and platelets or activate the complement system, ultimately leading to the destruction of blood cells [3, 11].
2. *Direct drug toxicity*: Piperacillin or tazobactam may directly act on bone marrow hematopoietic stem cells, inhibiting their proliferation and differentiation, resulting in a decrease in the production of neutrophils and platelets [12].
3. *Individual differences*: Age, underlying diseases, cumulative dose, duration of treatment, and concomitant medications may all affect the metabolism of PIP‐TAZ, potentially leading to neutropenia and thrombocytopenia in some patients [13].
4. *Consider the potential interaction between chronic renal failure and PIP‐TAZ*: The patient's chronic renal failure may have increased her risk of this adverse reaction. Impaired renal clearance could lead to higher circulating levels of PIP‐TAZ, thus increasing the risk of bone marrow suppression [14].

To prevent hematological adverse reactions such as neutropenia and thrombocytopenia caused by PIP‐TAZ, we recommend the following preventive measures:

1. *Pre‐treatment evaluation*: Carefully inquire about the patient's medical history to determine if they have a history of allergies, hematological diseases, or other risk factors.
2. *Close monitoring*: Regularly monitor complete blood count (CBC), especially neutrophil and platelet counts, to detect any abnormal changes in a timely manner.
3. *Individualized medication*: Choose an appropriate dose and duration of treatment based on the patient's condition to avoid unnecessary long‐term use or high‐dose use.
4. *Be vigilant for adverse reactions*: Be aware of the potential adverse reactions of PIP‐TAZ and remain vigilant for their occurrence.

If adverse reactions are suspected, the medication should be discontinued immediately. Symptomatic treatment should be provided based on the specific situation, such as administering granulocyte colony‐stimulating factor (G‐CSF) and platelet transfusions.

It is also necessary to rule out other diseases that may cause neutropenia and thrombocytopenia, such as severe infection and DIC. Additionally, continue to monitor CBC after discontinuing the medication until it returns to normal.

This case underscores critical implications for clinical practice. First, PIP‐TAZ‐induced cytopenias may occur rapidly, even with short‐term therapeutic dosing, necessitating heightened vigilance in high‐risk populations such as elderly patients or those with renal impairment. Second, the delayed recovery of platelet counts despite transfusions highlights the importance of early drug discontinuation over supportive measures alone. Clinicians must recognize that neutropenia and thrombocytopenia are not exclusive to prolonged or high‐dose antibiotic use. Finally, this case reinforces the need for individualized risk–benefit assessments when prescribing broad‐spectrum antibiotics like PIP‐TAZ, particularly in patients with limited therapeutic alternatives. These findings align with prior studies reporting hematological toxicity in PIP‐TAZ‐treated patients with renal dysfunction, suggesting that impaired drug clearance may amplify risks. Proactive monitoring, early recognition, and alternative antibiotic selection could mitigate life‐threatening complications in similar cases.

## 6 Preventative Recommendations

1. *Risk stratification*: We discuss the importance of identifying patients at increased risk of developing thrombocytopenia, including those with chronic kidney disease, a history of drug allergies, or underlying hematological conditions.
2. *Monitoring strategies*: We outline recommendations for monitoring platelet counts and other relevant laboratory parameters during PAP‐TAZ treatment, emphasizing the need for close monitoring in high‐risk patients.
3. *Alternative treatment considerations*: We discuss potential alternative treatment options for patients who are at high risk of developing thrombocytopenia or who have experienced this adverse effect in the past.
4. *Additional preventative measures*: We explore other potential preventative measures, such as adjusting the dosage of PAP‐TAZ based on renal function and considering the use of prophylactic platelet transfusions in high‐risk patients.

This report, based on a single case without a control group or in‐depth investigation, is insufficient to draw generalizable conclusions about the incidence and risk factors associated with PIP‐TAZ‐induced neutropenia and thrombocytopenia. The report only focuses on the patient's short‐term condition before discharge, lacking long‐term follow‐up data. Future research should involve larger studies, investigation of mechanisms, and identification of high‐risk populations to develop more effective prevention and monitoring strategies.

In summary, our case report highlights the potential risks associated with PIP‐TAZ.

When using this medication, clinicians should closely monitor the patient's CBC and be vigilant for potential hematological adverse reactions. By taking effective preventive and management measures, we can reduce the risk of such adverse reactions and ensure patient safety.

## 7 Conclusion

This case report highlights the potential for piperacillin/tazobactam to cause neutropenia and thrombocytopenia, emphasizing the need for close monitoring, prompt recognition, and timely intervention in patients with chronic diseases or renal insufficiency. Clinicians should prioritize regular CBC monitoring, especially in high‐risk populations, and consider alternative antibiotics in patients with preexisting renal insufficiency or hematological vulnerabilities.

## Author Contributions

**Hongyu Zhang:** conceptualization, data curation, formal analysis, investigation, methodology, writing – original draft. **Kaile Wu:** formal analysis, funding acquisition, investigation, software. **Ka Liang:** formal analysis, methodology, project administration, software, supervision, validation. **Zheng Peng:** writing – review and editing. **Ping Yi:** writing – review and editing, supervision.

## Ethics Statement

The publication of this report has been conducted with the proper consent of the patient, and the patient has personally signed a written informed consent form, ensuring the legality of the information and the protection of privacy.

## Conflicts of Interest

The authors declare no conflicts of interest.

## Data Availability

The data that support the findings of this study are available from the corresponding author upon reasonable request.
